# Positive health beliefs are associated with improvement of glycated hemoglobin and lipid profiles in Mexican patients with type 2 diabetes mellitus: a cross-sectional study

**DOI:** 10.1186/s12889-020-08866-4

**Published:** 2020-05-24

**Authors:** Abril Violeta Muñoz-Torres, Patricia Medina-Bravo, Brenda Elizabeth Valerio-Pérez, Grecia Mendoza-Salmeron, Jorge Escobedo-de la Peña, Lubia Velázquez-López

**Affiliations:** 1 Department of Public Health, School of Medicine, National Autonomous University (Universidad Nacional Autónoma de Mexico, UNAM), Mexico City, Mexico; 2 Department of Endocrinology, “Federico Gómez” Children’s Hospital of Mexico, Ministry of Health (SSA), Mexico City, Mexico; 3 Department of Nutrition and Health, Iberoamerican University (Universidad Iberoamericana), Mexico City, Mexico; 4 Clinical Epidemiology Research Unit, “Carlos Mac Gregor Sánchez Navarro” Hospital, Mexican Social Security Institute (Instituto Mexicano del Seguro Social, IMSS), Mexico City, Mexico

**Keywords:** Health beliefs, Type 2 diabetes, Diet, Physical activity, Metabolic control

## Abstract

**Background:**

Health beliefs are relevant to consider in patients with type 2 diabetes since disease control depends mainly on the patient’s behaviour. The aim of this study was to assess the association between health beliefs and glycated hemoglobin levels in Mexican patients with type 2 diabetes.

**Methods:**

An analytical cross-sectional study was conducted, and 336 patients were included. Fasting blood levels of glycated hemoglobin, glucose, cholesterol; triglycerides, high-density lipoprotein cholesterol (HDL-c), and low-density lipoprotein cholesterol (LDL-c) were measured. Body fat percentage, weight, height; waist circumference, and systolic and diastolic blood pressures were also obtained. A previously validated self-administered questionnaire was used to assess the health beliefs with regards to non-pharmacological treatment. Health beliefs were classified as positive, neutral, and negative.

**Results:**

The average age of patients was 54.7 ± 8.5 years, with a higher proportion of females (69%). The questionnaire had a good internal consistency with a Cronbach’s alpha score of 0.83. More than 90% of patients attributed a health benefit to diet and exercise, 30 to 40% experienced barriers, and more than 80% had a perception of complications associated to uncontrolled diabetes. Patients with positive health beliefs had lower HbA1c levels (8.2 ± 1.7%) compared to those with neutral (9.0 ± 2.3%), or negative (8.8 ± 1.8%; *p* = 0.042).

The LDL-c levels were lower (*p* = 0.03), and HDL-c levels were higher (0.002) in patients with positive heath beliefs.

**Conclusions:**

Positive health beliefs are associated with better metabolic control indicators in patients with type 2 diabetes.

## Background

Type 2 diabetes is considered a public health problem, and it has been estimated that by 2040 there will be approximately 640 million people with this disease [1]. Type 2 diabetes affects the economy, social environment, and the quality of life of afflicted individuals [2].

Adequate management of the disease and the achievement of metabolic goals requires the active participation of patients in their own care. Diabetes education, diet, and physical activity all support the treatment and condition of the disease as well as the prevention of complications [3, 4]. There are different theoretical models related to the reasoned action and planned behavior in health care [5, 6]. According to Rosenstock, health care depends on the value credited to an action and to the perception of the individual on it producing a positive result [7]. The dimensions of the beliefs in health care model include: a) perceived susceptibility for getting sick, b) the severity of the subject’s perception that their physical health can be damaged and their social lives affected, c) the benefits they attribute to the change in behavior to improve their health condition, and, d) the barriers that impede the subject from following certain conduct in the care of their health [8].

According to this theoretical model, the actions for the attention, care, and prevention of the complications of diabetes will depend on the beliefs that the patient attributes to the benefits of care in their illness, to the risks, and to their perception of the severity of their diabetes.

In patients with type 2 diabetes, it has been reported that health beliefs are related to treatment adherence and attitudes towards care [9, 10]. In addition, some barriers have been identified that limit adherence to favorable lifestyle changes and care, as they are not considered relevant for achieving goals in controlling diabetes [11, 12].

The Mexican Social Security Institute (known as the *IMSS*) is the institution that provides medical care to nearly half of the Mexican population (includes private-sector employees and their family members, students, and habitants of rural communities) [13]. The *IMSS* provides medical care to 4.2 million patients with diabetes, and in 2017 the *IMSS* had 14.5 million medical consultations at the primary care level, and there were 600,000 hospitalizations due to complications of the disease [14].

In patients with type 2 diabetes in Mexico the lack of metabolic control is a challenge. According to the National Health and Nutrition Survey (ENSANUT) in 2012 only 24.5% of those with a previous diagnosis of diabetes had metabolic control [15, 16]. In patients with diabetes, there is limited evidence about their perceptions on the risk of complications, the benefits of care, and the barriers to adopting healthy lifestyles [17]. Educational strategies supported by theoretical models in patients with type 2 diabetes, which include more physical activity, have been shown to be useful in promoting adherence to treatment and diabetes care [18, 19].

It has been demonstrated that health beliefs related to diet, physical activity, food types and carbohydrate content of foods, are related to the occurrence of diabetes [20, 21].

In Mexico, there are deep-rooted cultural behaviours and health beliefs, and their identification in patients with diabetes can support the planning of educational strategies.

The importance of health beliefs in the care of the disease has been described; however, there is limited information about its impact on indicators of metabolic control. The objective of this study was to assess the relationship between health beliefs in diabetes and the glycated hemoglobin levels, lipid profiles, and body weight in Mexican patients with type 2 diabetes.

## Methods

### Study design and population

An analytical cross-sectional study was conducted, and 336 patients with type 2 diabetes were included. The Ethics and Research Committee of the institution approved the study. The researchers followed the World Medical Association International Code of Medical Ethics and the Declaration of Helsinki for experiments involving humans [22].

The study was conducted between March 2016 and September 2017. A sample size of 270 participants was estimated to identify at least 37% of participants with with neutral and negative health beliefs: the sample size calculation was performed with a 95% confidence interval (CI) and a 2% precision, and the sample size of 270 participants was obtained.

At the *IMSS* there are three identified levels of health care: the primary level comprises outpatients seeking medical care, drug prescriptions, nutritional care, nursing, blood and urine tests, and preventative procedures; the secondary level entails specialized care in regional hospitals where the patients are derived from primary care physicians, either for outpatient consultation or hospitalization; and, the tertiary level of care refers to specialized medical centers.

For the purpose of the present study, four primary care or family medical units were selected from the central and southern municipalities of Mexico City. All patients derived from these selected clinics sent to the hospital where the research was conducted, were invited to participate in the study by a nurse and a nutritionist; and those who accepted the invitation to participate were enrolled in the study. All patients were interviewed by a physician, a nutritionist, and a nurse who explained the objectives, benefits, and risks of the study. After any doubts and concerns were clarified, and participants voluntarily accepted to participate in the study, an informed consent was signed.

#### Eligibility criteria of the participants

Patients with at least 1 year of diagnosed type 2 diabetes and currently receiving treatment were recruited. Patients with a previous diagnosis of advanced diabetic retinopathy or blindness, severe diabetic neuropathy, diabetic foot, or end-stage renal disease were excluded.

A clinical exam was performed by a participating physician to retrieve data regarding socio-demographics, treatment, clinical history, and any comorbidities.

#### Clinical measurements

Blood pressure was measured on two occasions with a mercury sphygmomanometer with an interval of 5 min between each measurement, after the patient had remained seated for at least 5 min. The average value was used for the analysis. Hypertension was identified by a previous diagnosis of hypertension and the use of antihypertensive drugs, and in those with two blood pressure measurements ≥140/90 mmHg.

#### Biochemical measurements

Glycated hemoglobin (HbA1c) was measured in venous blood by high performance liquid chromatography (HPLC) after fasting for 12 h. Glucose, creatinine, triglycerides; total, high-density (HDL-c), and low-density lipoprotein cholesterol (LDL-c) were measured using the method of automated photometry (Roche Cobas 800 c701).

#### Measurements of anthropometry and body composition

Certificated nutritionists recorded the anthropometric measurements using the method proposed by Habitch and the specifications recommended by Lohman et al. [23, 24]. Weight and height were assessed, and the body mass index (BMI) was estimated. Waist circumference (WC) was measured after determining the midpoint between the last rib and the upper edge of the iliac crest on the right side, while the hip circumference was measured at the greatest diameter of the trochanters. Both measurements were taken three times with the average value of the second and third measurements used for the analysis. The percentages of fat and fat mass were obtained through bio-impedance of the lower segment using a TANITA™ analyzer model TBF-215.

#### Other measurements

When the subject mentioned having received any formal advice on diabetes either by a physician, nurse, or a nutritionist, they were considered as having received previous diabetic education. Formal diabetes education at the *IMSS* includes twelve modules in educational sessions. The modules were reviewed each month (one module per month) in 60-min educational sessions [25]. When the subject had received personalized nutritional guidance by a nutritionist, they were considered to have received previous nutritional therapy.

Physical activity was considered when the patient complied with the guidelines of the World Health Organization (WHO) in this area, having had regular exercise in the previous month with moderate intensity ≥150 min or vigorous intensity ≥75 min per week [26].

#### Health beliefs measurements

##### Instrument selection

A bibliographic review of previous instruments that evaluated health beliefs in patients with diabetes was performed. Studies that were easily understood and applied in the clinical setting in patients with type 2 diabetes, were included in the review. The evaluation of health beliefs in patients with diabetes identified by other authors was revised and adapted into a new questionnaire, including general concepts of diabetes, diet, and exercise [27, 28].

##### Content validation

The first version of the self-administered questionnaire was developed to allow for the measurement of health beliefs in patients with type 2 diabetes, including diet and physical activity.

The logical and content validity was blindly evaluated in a consensus of seven diabetes experts including two nutritionists, a psychologist, two physicians, and two clinical researchers. Evaluation included assessing the level of comprehension and readability of the instrument. Three items were modified in this step.

After a pilot test in 32 patients with diabetes from a primary care clinic, 8 items of the instrument were eliminated due to the lack of variability in the responses or difficulty in understanding according to the criteria of the experts. In the end, the instrument included 15 items. The final instrument included four domains based on the health beliefs theory. The instrument included health beliefs related to the susceptibility (items 1 and 5), the severity of diabetes (items 2 to 4), the benefits of preventive actions (items 6,8,9,11,14), and beliefs related to the barriers (items 7,10,12,13,15).

The score for each item ranged from 0 to 4. Health beliefs were then classified as positive when the score was (≥48), neutral (39 to 47), or negative (≤38). The instrument and score is shown in the supplemental appendix 1.

For qualification of the susceptibility domain, the values ranged from positive health (7 to 8), neutral (4 to 6), and negative (≤3). Beliefs on severity were classified as positive (9 to 12), neutral (5 to 8), and negative (≤4). Values in the benefits and complications domain were classified as positive (17 to 20), neutral (13 to 16), and negative (≤12).

Cronbach’s alpha analysis of the instrument used in the population with type 2 diabetes in this study was performed (*n* = 336). A value of 0.83 was obtained, and this value is considered reliable because it has a good internal consistency of the instrument being applied [29].

### Statistical analysis

The statistical analysis was performed with the statistical package IBM SPSS Desktop 22.0. Measures of central tendency and dispersion were estimated for age, duration of disease, blood pressure, and metabolic control parameters: weight, BMI, WC, HbA1c, glucose, and lipid profiles. Measures of frequency and percentages were used for categorical variables including the health beliefs domains. The internal consistency of the questionnaire implemented in the study was determined by the Cronbach reliability coefficient (Cronbach’s alpha).

To compare the difference between the levels of the metabolic control indicators according to the type of health belief identified (positive, neutral, and negative health beliefs), the unidirectional analysis of variance test (ANOVA) was performed. A linear regression model was employed considering the dependent variable as HbA1c. The model was adjusted by total score of the health beliefs instrument, age, years since diagnosis, and sex. A value of *p* < 0.05 was considered statistically significant.

## Results

The sociodemographic characteristics and comorbidities of the population studied are described in Table 1. A total of 336 patients were included in the study. The average age was 54.7 ± 8.5 years, with a higher proportion being female (69%). The average HbA1c was 8.2 (7.1–10.3%). In the lipids profile, only total cholesterol was found within acceptable range (median 195, range 172–218 mg/dL). In anthropometric data, the average BMI stands out (30.3 ± 5.1 kg/m^2^). Oral hypoglycemic drugs are the most widely used in treating diabetes. The median number of years since diagnosis with the illness was 6 years (range 3.0–11.0 years). Positive beliefs were identified only in 21% of the population studied.

**Table 1:** Sociodemographic characteristics and comorbidities in patients with type 2 diabetes. ***n*** **= 336**

	**Frequencies and percentages**
Women	230 (69)
Diabetes education	98 (29)
Nutrition therapy	117 (35)
Physical exercise	52 (15)
Smoker	163 (48)
Alcohol consumption	138 (41)
Educational Level	
Primary	184 (54)
High School	103 (31)
Post-secondary	49 (15)
Hypertension	150 (45)
*Diabetes treatment*	
Hypoglycemics	243 (72)
Hypoglycemic/ insulin	42 (13)
Insulin	36 (11)
No drugs	15 (4)
*Health Beliefs*	
Positive	71 (21)
Neutral	107 (32)
Negative	158 (47)
	**Medians and interquartile ranges (Q1-Q3)**
Years with diabetes diagnosis	6.0 (3.0–11.0)
HbA1c (%)	8.2 (7–10)
Glucose (mg/dL)	152.0 (125–201)
Creatinine (mg/dL)	0.77 (0.70–0.87)
Total cholesterol (mg/dL)	195 (172–218)
LDL-c (mg/dL)	113(93–134)
HDL-c (mg/dL)	
Women	42 (36–48)
Men	37 (31–41)
Triglycerides (mg/dL)	178(135–243)
	**Mean and Standard deviation**
Age (years)	54.7 ± 8.5
Weight (Kg)	74.7 ± 14.4
BMI (Kg/m^2^)	30.3 ± 5.1
WC (cm)	
Women	99.8 ± 12.3
Men	100.9 ± 12.3
Fat (%)	42.0 ± 11.8
Fat mass (kg)	32.3 ± 13.1
SBP (mmHg)	124.8 ± 14.4
DBP (mmHg)	83.5 ± 10.1

*SBP* Systolic blood pressure. *DBP* Diastolic blood pressure. *LDL-c* Low Density Lipoproteins cholesterol. *HDL-c* High Density Lipoproteins cholesterol. *BMI* Body Mass Index. *WC* Waist Circumference

Table 2 shows data from the health beliefs instrument. Only 30% considered that their disease was controlled, while 93% believed that physical activity conferred benefits to prevent complications, and 94% believed that they could develop complications with inadequate nutrition. In total, 84 to 94% of patients perceived the complications and severity of the disease. Barriers to the adherence to diet and exercise regimens persisted in 60 to 70% of the sample, and 77% believed their family supported them in their efforts to adhere to a healthy diet.

**Table 2:** Distribution of responses to the health belief instrument in patients with type 2 diabetes T2DM. *n* = 336

Items	n (%)		
	Agree	Not sure	Disagree
1. I think my diabetes is controlled.	99 (30)	156 (46)	81 (24)
2. I think that if my glucose is out of control I have the risk of suffering from some complication of diabetes.	275 (82)	34(10)	27 (8)
3. I think that if I have any complication of diabetes it could be serious.	299 (89)	25 (7)	12 (4)
4. I believe that being overweight puts me at risk for the complications of diabetes.	312 (92)	12 (4)	12 (4)
5. I believe that having high blood cholesterol can cause health problems.	308 (92)	22 (6)	6 (2)
6. I consider that I follow a diet that allows me to take care of my diabetes.	125 (37)	131 (39)	80 (24)
7. I think it is very difficult to reach a healthy weight.	155 (46)	82 (24)	99 (30)
8. I think that a poor diet is likely to lead to the development of diabetes complications.	317 (94)	11 (4)	8 (2)
9. I believe that exercise helps take care of my glucose levels (blood sugar).	314 (93)	16 (5)	6 (2)
10. I think I would lose weight if I had fewer occupations in the day.	102 (30)	98 (29)	136 (41)
11. I think that due to lack of exercise I can develop complications of diabetes.	281 (84)	33 (10)	22 (6)
12. I believe that because of my daily activities it is difficult for me to follow an exercise program.	149 (44)	61 (18)	126 (38)
13. I believe that due to lack of time it is difficult for me to stick to a diet for my diabetes.	165 (49)	61 (18)	110 (33)
14. I consider that I have the support of my family to follow a diet that controls my diabetes.	258 (77)	38 (11)	40 (12)
15. I think I need more income to follow a diet that controls my diabetes.	152 (45)	65 (19)	119 (35)

The data are presented as frequencies and percentages

Patients with positive health beliefs had lower HbA1c levels (8.2 ± 1.7%) than those with neutral health beliefs (9.0 ± 2.3%) or negative health beliefs (8.8 ± 1.8%; *p* = 0.04). The LDL-c levels were also lower in patients with positive heath beliefs (*p* = 0.03), and HDL-c levels were higher in the group with positive health beliefs (*p* = 0.002). The total cholesterol levels were lower in neutral group. Data are shown in Table 3.

**Table 3:** Mean values of metabolic control parameters according to health beliefs. *n* = 336

Biochemical indicators	Positive ^**a**^ 71 (21%)	Neutral ^**b**^ 107(32%)	Negative ^**c**^ 158 (47%)	*p* value*
HbA1c %	8.2 ± 1.7 ^b^	9.0 ± 2.3 ^a^	8.8 ± 1.8	0.042
Fasting glucose mg/dL	159.8 ± 52.1	183.0 ± 82.1	169.3 ± 59.3	0.061
Total cholesterol mg/dL	199.4 ± 34.0	189.1 ± 41.0 ^c^	203.0 ± 43.6 ^b^	0.025
LDL-c mg/dL	115.0 ± 28.7	106.9 ± 31.8 ^c^	117.0 ± 32.3 ^b^	0.035
HDL-c mg/dL	43.5 ± 11.6 ^b^	38.3 ± 8.6 ^a, c^	42.5 ± 12.1 ^b^	0.002
Triglycerides mg/dL	188.3 ± 78.1	213.7 ± 115.5	204.5 ± 102.3	0.270
Weight kg	71.9 ± 12.5	76.4 ± 14.9	74.8 ± 14.8	0.129
BMI kg/m ^*2*^	29.3 ± 4.1	30.6 ± 5.4	30.5 ± 5.3	0.220
Waist circumference cm	97.8 ± 9.1	101.5 ± 14.0	100.2 ± 12.3	0.147
Fat %	42.1 ± 11.0	42.1 ± 12.8	41.8 ± 11.6	0.973
SBP mmHg	122.1 ± 12.6	125.8 ± 16.1	125.4 ± 13.9	0.185
DBP mmHg	81.9 ± 9.9	84.1 ± 11.0	83.8 ± 9.6	0.337

The data are as mean ± SD. *SBP* Blood Pressure Systolic; *DBP* Blood Pressure Diastolic. *LDL-c Low Density Lipoproteins cholesterol. HDL-c High Density Lipoproteins cholesterol. BMI Body Mass Index. WC Waist Circumference*

* *p* value was calculated with ANOVA test

^a, b, c^ The letters indicate which groups show a statistically significant difference in each indicator

Health beliefs domains and their relation to HbA1c are shown in Table 4. Patients who perceived the domain of susceptibility to a complication of diabetes in a positive category, were associated with a lower HbA1c level.

**Table 4:** Mean HbA1c values according to health belief domains and instruments responses. *n* = 336

	n (%)	HbA1c %*	***p*** value
**Susceptibility**			
Positive ^a^	91 (27.1)	8.1 ± 1.7 ^b, c^	0.020
Neutral ^b^	235 (69.9)	8.9 ± 2.1 ^a^	
Negative ^c^	10 (3.0)	9.7 ± 2.2 ^a^	
**Severity**			
Positive	285 (84.8)	8.7 ± 2.0	0.541
Neutral	43 (12.8)	9.1 ± 2.1	
Negative	8 (2.4)	8.5 ± 1.9	
**Benefits**			
Positive	170 (50.6)	8.7 ± 2.1	0.944
Neutral	136 (40.5)	8.7 ± 1.9	
Negative	30 (8.9)	8.8 ± 1.9	
**Barriers**			
Positive	45 (13.4)	8.7 ± 1.9	0.171
Neutral	51 (15.2)	8.2 ± 1.8	
Negative	240 (71.4)	8.8 ± 2.0	

Data are presented as mean and standard deviation, *p* value was calculated with ANOVA test

^a,b,c^ The difference in the letters indicate a statistically significant difference between categories

The results of the multivariate model, presented in Table 5, show that when the score of the patient’s health beliefs is lower there is an increased risk of having a higher HbA1c levels. A longer time since diagnosis of diabetes also influences this result.

**Table 5:** Health beliefs and other factors associated with HbA1c values in a multivariate linear regression model in patients with type 2 diabetes. *n* = 336

Variables	Coefficients	t	*p* value	95% CI (lower)	95% CI (upper)
Health beliefs (score)	−0.038	−2.56	0.011	−0.068	− 0.009
Age (years)	−0.053	−4.07	0.001	−0.077	− 0.027
Sex	0.281	1.225	0.221	−0.170	0.733
Diabetes diagnosis (years)	0.066	3241	0.001	0.026	0.107
Nutrition therapy	0.395	1.763	0.079	−0.046	0.836
Constant	11.68	10.43	0.001	9.47	13.88

Model adjusted for age, sex, years of diabetes diagnosis and nutrition therapy

95% C.I. (Confidence Interval)

## Discussion

The treatment of type 2 diabetes requires the active participation of the patient in the management of their disease. Adherence to pharmacological treatments and to following healthy lifestyles depends on the patient’s perception of barriers, benefits of diet and exercise, and the risk of complications.

Our results show that health beliefs are directly associated with metabolic control in patients with diabetes. Patients with positive health beliefs have better metabolic control. Health care procedures focused on the beliefs and culture of the population are therefore relevant. It has even been shown that for patients with diabetes educational programs that take their culture into account there are beneficial effects on glycemia control, knowledge about diabetes, and maintenance of a healthy lifestyle [30]. Nevertheless, health beliefs can span several generations, as seen in the preference for natural treatments, underestimation of the importance of the disease and its treatment, and the poor perception regarding the occurrence of complications in the absence of proper health care [31, 32].

Prior to providing diabetes education, it is necessary to identify the health beliefs in patients with diabetes so that education can focus on improving knowledge, removing barriers, and influencing healthy behaviors for diabetes care. Other authors have observed that for diabetes patients interventions that take into account the ethnicity and culture of the patient can provide positive effects in improving glycemia control, knowledge of type 2 diabetes, and changes in lifestyles [30, 33].

Also, even though 84% of the patients studied perceive the severity by which they can develop a complication of diabetes, this is not associated with a better HbA1c level. It has been shown that educational strategies may not influence the improvement of metabolic control but that the strategies may increase perception of the risk of complications [34]. Again, in the domain of susceptibility, patients who perceived a complication of diabetes in a positive way were associated with a lower HbA1c level.

This finding is relevant since it should encourage educational strategies to reinforce the importance of a healthy diet and physical activity to increase the patient’s perception of their role in treatment to attain metabolic control. Health beliefs should be considered to diminish perceived barriers and to improve adherence to both pharmacological and non-pharmacological treatments [35]. Despite a positive perception of the importance of complications, and the benefits attributed to healthy diet and physical activity, there are still barriers to treatment compliance. There is a need to develop educational strategies that include family members in order to overcome real or perceived barriers [36, 37]. Since dyslipidemia in diabetes is a known cardiovascular risk factor, better lipid profiles in those with positive health beliefs is undoubtedly an important finding [38].

It is necessary to reinforce health professional and family supports for better adherence to healthy lifestyles. Recently, it was identified that the patient will take greater care of their disease and adhere better to their diet if they perceive a benefit from and the support of their physician [39]. Different beliefs and barriers to self-management in the care of the disease have been previously described in Hispanics with lower income, lower education, and less knowledge of diabetes [40, 41]. It is also important to note that the mean HbA1c in the population studied was higher than the goal value, similar to the 10.6% HbA1c mean value reported by the National Health and Nutritional Survey in Mexico [42].

Barriers to adherence to a healthy lifestyle persist in health beliefs, since only 29% reported they did not have barriers to achieving a healthy diet or performing physical exercise. Similar results were reported in a review of such barriers where economic status and barriers to communication with the physician primarily stood out, although little research on the characteristics of the affected patients has been performed [43]. These results are similar to those previously described where relevant aspects associated with culture, family support, and poor disease knowledge were substantiated [44]. It has been previously reported that limited adherence to a healthy diet or physical activity persists in the treatment in patients with type 2 diabetes [45]. Additionally, patients with diabetes in Mexico continue to suffer from obesity, dyslipidemia, and uncontrolled glycemia, and they have a greater risk of experiencing complications. It is thus imperative to incorporate preventive measures to diminish these risk factors [46, 47]. It is vital to insist that patients incorporate diet and physical exercise as part of their integral treatment, and future studies should consider these aspects to motivate the patient to perform the appropriate actions to manage their illness [48].

Patients may perceive benefits of diet and physical exercise and are aware of the complications that can arise due to poor management of their disease, yet barriers to adhering to non-pharmacological treatment also persevere; therefore, patients continue to suffer from poor metabolic control and an increased risk of vascular complications [49].

The strengths of the present study stem from the construction of an instrument by clinical experts and the analysis of internal consistency, in obtaining a reliable instrument. Our results are consistent with other authors who have reported the perception of the risk of prediabetes and diabetes in patients, and the importance of following a diet and doing physical activity. Even so, the patients had barriers to improve their health and had a lower perception of the benefits of adhering to their treatment [27, 28].

Among the limitations of the present study, is the type of study design that did not permit robustly establishing a causal relationship between health care beliefs and their impact on metabolic control. Also, the health beliefs identified were from diabetic patients in an urban zone of Mexico, and it would be necessary to replicate the study in other geographical regions to identify the external validity of the instrument. Another important aspect is that more females were included in the study (69%), and it would be worthwhile to see, in future studies, a greater participation of males, given that the management of the illness could differ due to different social, work, and family networks. One other possible limitation of the study is that the recording of nutritional therapy, diabetes education, or physical activity was only obtained through patient interrogation. Future studies should consider socio-economic and family status in the relationship between health care beliefs and metabolic control, among other variables that can affect health care and metabolic control.

## Conclusion

Positive health beliefs in relation to non-pharmacological treatment are associated with improved HbA1c levels and lipid profile indicators. Only 21% of interviewed patients had positive health beliefs. Patients with type 2 diabetes continue to exhibit risk factors such as obesity, dyslipidemia, and lack of glycemia control. It is very important to insist on educational strategies that consider the health beliefs of patients in order to promote general care in diabetes, a healthy diet, and physical exercise as integral to the treatment of their illness.

## Data Availability

The data sets used and/or analyzed during the current study are available from the corresponding author upon reasonable request.

## Appendix

**Table 6:** Instrument of health beliefs for patients with type 2 diabetes type 2 diabetes mellitus

	Agree	I’m not sure	Disagree
1. I think my diabetes is controlled.	4	2	0
2. I think that if my glucose is out of control I have the risk of suffering from some complication of diabetes.	4	2	0
3. I think that if I have any complication of diabetes it could be serious.	4	2	0
4. I believe that being overweight puts me at risk for the complications of diabetes.	4	2	0
5. I believe that having high blood cholesterol can cause health problems.	4	2	0
6. I consider that I follow a diet that allows me to take care of my diabetes.	4	2	0
7. I think it is very difficult to reach a healthy weight.	0	2	4
8. I think a poor diet is likely to lead to the development of diabetes complications.	4	2	0
9. I believe that exercise helps take care of my glucose levels (blood sugar).	4	2	0
10. I think I would lose weight if I had fewer occupations in the day.	1	2	3
11. I think that due to lack of exercise I can develop complications of diabetes.	4	2	0
12. I believe that because of my daily activities it is difficult for me to follow an exercise program.	0	2	4
13. I believe that due to lack of time it is difficult for me to stick to a diet for my diabetes.	0	2	4
14. I consider that I have the support of my family to follow a diet that controls my diabetes.	4	2	0
15. I think I need more income to follow a diet that controls my diabetes.	0	2	4

The score for each item ranged from 0 to 4. Health beliefs were positive (> 48), neutral (39 to 47), negative (< 38)

## Abbreviations

HbA1c: Glycated hemoglobin
HDL-c: High-density lipoprotein cholesterol
LDL-c: Low-density lipoprotein cholesterol
HPLC: High performance liquid chromatography
BMI: Body Mass Index
*IMSS*: Mexican Social Security
*INEGI*: Institute Intercensal Survey by the National Institute of Statistics and Geography
ENSANUT: National Health and Nutrition Survey ANOVA: Analysis of variance
Q: Quartile
CI: Confidence Interval
